# A multilevel analysis of improved drinking water sources and sanitation facilities in Ethiopia: Using 2019 Ethiopia mini demographic and health survey

**DOI:** 10.3389/fpubh.2023.1063052

**Published:** 2023-02-14

**Authors:** Jember Azanaw, Eshetu Abera, Asmamaw Malede, Mastewal Endalew

**Affiliations:** ^1^Department of Environmental and Occupational Health and Safety, Institute of Public Health, College of Medicine and Health Sciences, University of Gondar, Gondar, Ethiopia; ^2^Department of Environmental Health, College of Medicine and Health Sciences, Wollo University, Dessie, Ethiopia

**Keywords:** access, improved water sources, improved sanitation, EDHS, Ethiopia

## Abstract

**Background:**

Access to water, sanitation, and hygiene is an important element for communicable disease control including the existing COVID-19 pandemic. This is due to the growing water demand and decreasing water availability, because of shrinking resources, increased urbanization, and pollution. This problem is higher, particularly among least developed countries like Ethiopia. This study, therefore, aimed at investigating the level of improved water sources and sanitation as well as their predictors in Ethiopia using EMDHS-2019.

**Method:**

Mini Ethiopian Demographic and Health Surveys 2019 database survey was used in this study. Data collection took place over 3 months, from 21 March 2019 to 28 June 2019. A total of 9,150 households were selected for the sample, of which 8,794 were engaged. Among involved households, 8,663 were successfully interviewed at a response rate of 99%. The dependent variables measured in this study were improved drinking water sources and sanitation facilities. Due to the nested nature of DHS data, multilevel binary logistic regression analysis was done using Stata-16.

**Results:**

The majority (72.62%) of household heads were men, and 69.47% of participants were from rural areas. Close to half (47.65%) of study participants did not have any form of formal education, while the lowest proportion (9.89%) of them had higher education. Approximately 71.74 and 27.45% of the households have accessed improved water sources and sanitation, respectively. Based on the final model results, wealth index, educational status, and having a television individual-level variables while community-level poverty, community-level education, community-level media exposure, and place of residence were statistically significant predictors of getting improved water source and sanitation.

**Conclusion:**

The level of access to improved water sources is moderate but it lacks progress, while access to improved sanitation was lower. Based on these findings, great improvements should be made in providing access to an improved water source and sanitation facilities in Ethiopia. Based on these findings, great improvements should be made in providing access to improved water source and sanitation facilities in Ethiopia.

## Background

The World Health Organization and United Nations Children's Fund (WHO/UNICEF) Joint Monitoring Programme for Water Supply, Sanitation and Hygiene (JMP) produces internationally comparable estimates of countrywide, regional, and worldwide development on drinking water, sanitation, and hygiene (WASH) and is accountable for international monitoring of the Sustainable Development Goal (SDG) targets related to WASH (1). SDG-6 aims to “ensure availability and sustainable management of water and sanitation for all and includes targets for universal access to safe drinking water (6.1), sanitation, and hygiene” (6.2) (2). Access to water, sanitation, and hygiene is an important element for communicable disease control (3), including the existing COVID-19 outbreak (4).

Globally, 1.8 billion people gained access to at least basic water services between 2000 and 2017. Despite this, in 2017, 2.2 billion individuals still lacked access to properly managed drinking water, 4.2 billion lacked safely managed sanitation, and 3 billion lacked basic hand washing facilities internationally (5). According to WHO/UNICEF, 2017 report only 24% of the rural population and 44% of the urban population have access to sanitation facilities in sub-Saharan African countries (6). This is due to the growing water demand and decreasing water availability, because of shrinking resources, urbanization, and pollution (7).

Insufficient drinking water, sanitation, and hygiene (WASH) are key predictors of numerous wide-ranging disease burdens, focusing primarily on diarrheal diseases in low-income settings (8). More than 50 pathogens that are accountable for diarrheal illness, schistosomiasis, and soil-transmitted helminth infections, communicated due to unsatisfactory sanitation (9). As well as access to safe water, sanitation and hygienic situations have a vital role in protecting human health from emerging and re-emerging disease outbreaks, including the existing COVID-19 pandemic (10).

At the same time, household water demand is estimated to increase significantly over the period 2010–2050 in all the globe except for Western Europe (7). The more carefully planned out drinking water and sanitation are in a country's national development goals, the more important these issues are likely to be to that nation's policymakers (2). However, the accessibility of water is endangered by climate change, population growth, changes in demographic characteristics, and urbanization (11).

Previous research has shown that in Africa some of the factors associated with access to improved household water sources include the place of residence, wealth status (12–14), education, ethnicity, access to electricity, gender, water collection time, and the number of rooms in a household (15). Due to these problems, the goals of the sustainability Development Strategy in achieving universal access to safe water and sanitation are still extremely far from its anticipated target, particularly among least-developed countries like Ethiopia (12). Every 5 years, DHS is conducted in Ethiopia which includes water and sanitation. According to research conducted in 2020 by Water.org, only 42% of the population in Ethiopia has access to clean water; of which, only 11% of that amount has access to sufficient sanitation services (16).

Continuous studies are needed until the occurrence of inequalities toward WASH is reduced and warrants sustainable development in unindustrialized nations. This could be used as evidence for any concerned body targeting that would possibly involve investment and resources to advance their access to water and sanitation to reach worldwide access by 2030. Practically, still there is a higher prevalence of open defecation and intermittent water supply in the towns and rural areas of the country. Even though there are few of these studies have done using such representative data to examine the magnitude and determinants of access to improved drinking water sources and sanitation, there is a need to do more research, especially during the time of COVID-19. Examining such patterns is helpful evidence for health-related policy makers, Non-governmental Organizations (NGOs), and all other stakeholders responsible for WASH. Therefore, this study was aimed at investigating the level of improved water and sanitation as well as their predictors in Ethiopia using EMDHS-2019.

## Methods

### Study setting and data source

This study was done in Ethiopia, which is the second-largest number population next to Nigeria in Africa. Mini Ethiopian Demographic and Health Surveys 2019 (EDHS-2019) database survey was used in this study. MEDH survey was conducted in nine geographical regions (Tigray, Afar, Amhara, Oromia, Somali, Benishangul-Gumuz, Southern Nations Nationalities and Peoples Region (SNNPR), Gambella, and Harari) and two administrative cities (Addis Ababa and Dire Dawa) of the country. This MEDHS Survey is a nationwide representative population-based survey with large sample sizes. EDHS data are open source and can be retrieved on the DHS website (https://dhsprogram.com/Data/terms-of-use.cfm).

The 2019 EMDHS sample was a two-stage stratified cluster sample, sampling weights were calculated based on sampling probabilities separately for each sampling stage and for each cluster. In the first stage, a total of 305 EAs (93 in urban areas and 212 in rural areas) were selected with probability proportional to EA size (based on the 2019 EPHC frame) and with independent selection in each sampling stratum. In the second stage of selection, a fixed number of 30 households per cluster were selected with an equal probability of systematic selection from the newly created household listing. EPHI investigators, an ICF technical specialist, an advisor, and representatives from other organizations, including CSA, FMoH, the World Bank, and USAID, supported the data collection. Data collection took place over a three-month period, from 21 March 2019 to 28 June 2019. A total of 9,150 households were selected for the sample, of which 8,794 were engaged. Among involved households, 8,663 were successfully interviewed at a response rate of 99%.

### Study variables

#### Outcome variables

Improved water sources were defined as water from piped water, boreholes or tube wells, protected dug wells, protected springs, rainwater, and packaged or delivered water (12, 17, 18). While access sanitation facilities, such as flush/pour flush to piped sewer systems, septic tanks, or pit latrines, were defined as improved sanitation facilities, ventilated improved pit latrines, composting toilets, or pit latrines with slabs were also included (12, 17, 18).

The dependent variables measured in this study were improved drinking water sources and improved sanitation facilities. Unimproved water sources or sanitation facilities were represented as dichotomous variables, with “1” representing “improved” and “0” representing “unimproved”, respectively, for both water sources and sanitation.

### Predictor variables

#### Individual-level variables

Sex of household head (male or female), wealth index (poor, middle, and rich), educational status, and having television and radio were individual predictor variables. The poorest and poor were coined as “poor”, middle as “middle” whereas rich and richest, were categorized as “rich”.

#### Community-level variables

Community-level education, the place of residence (urban/rural), community-level media exposure, region [(recoded as pastoralist region (Benishangul, Somali, Gambella, and Afar), Semi-pastoralist (Oromia, SNNPR), Agrarian (Amhara and Tigray) and City administration (Addis Ababa, Dire Dawa, and Harari)], community-level educational attainment, community-level poverty, and community-level media exposure were community-level variables. All the variables were selected using previous related literature review (19–22).

### Data quality assurance

For data quality assurance purposes, the 2019 EMDHS pretest containing in-class training, biomarker training, and field exercise days was done. The field exercise was conducted in clusters around Adama, which were not included in the 2019 EMDHS sample. A debriefing session was held with the pretest field staff, and adjustments to the questionnaires were done based on lessons drawn from the field practice. Trained collectors, supervisors, field editors, interviewers, secondary editors, and reserve interviewers were engaged to correct the limitation of the tool during the pretest.

### Operational definition

#### Improved water on premises

Having access to improved water sources located at 0 min from the point of use (23, 24).

#### Basic water service

Having access to an improved water source located between 1 and 30 min, for a roundtrip (23–25).

#### Limited water service

Having access to improved water sources located farther than 30 min for a round trip (23–25).

#### Unimproved water sources

This refers to all unimproved water sources irrespective of the collection time (23, 24).

#### Improved sanitation

Confirm hygienic separation of human feces from human contact using flush/pour flush to the piped sewer system, septic tank, pit latrine, ventilated improved pit (VIP) latrine, pit latrine with slab, and composting toilet (26).

### Data analysis

Due to the nested nature of DHS data, multilevel binary logistic regression analysis was done. Descriptive analysis was employed to examine the frequency, percentage, mean, and standard deviation of the variables of interest. Subgroup analysis was done by splitting the data by geographic location, educational status, and wealth status of the participant to make comparisons among subgroups in the data. Bivariable and multivariable multilevel binary logistic regression was utilized to evaluate associations between outcome variables and independent variables because the outcome variables were dichotomous (improved and unimproved). Independent individual variables in bivariable analysis with a *p*-value <0.2 were included in multivariable multilevel logistic regression analysis. Then 95% confidence interval (CI) was employed, and a *p*-value <0.05 was used for testing statistical significance in multivariable logistic regression analysis.

Before undertaking the logistic regression, the variables were tested for multicollinearity using the method of forward and backward correlation. Considering that the EDHS survey used complex sampling, sampling weights were applied to each analysis in order to adjust the variances in the probability of sample selection.

Four models comprising important variables were built-in in this study. These models were run individually and aimed at accessing improved water sources and improved sanitation services. Model 0 (Null model) was fitted without independent variables to test random variability in the intercept and to estimate the mean odds ratio (MOR), intra-class correlation coefficient (ICC), and proportion change in variance (PCV). Model II measured individual-level variable effects on the outcome variables. Model III evaluated the effects of community-level factors on dependent variables. Model IV (final model) is used to determine the effects of individual and community-level variables instantaneously dependent variables.

ICC, MOR, and PCV were calculated as follows:


$$ICC=\frac{Va}{Va*\;(\pi^{2})/3}=\frac{Va}{\;(Va*3.29)}$$

where Va is the area-level variance.

PCV = ${\frac{Vc-Vl}{Vc}}^{*}100\%$, where Vc was the variance of the lowest model, and Vl was the variance of the model with more predictor variables.

MOR = $\exp{\left[\sqrt{\;(2^{*}Va\;}\right)}^{*}0.6745]$ ≈ $\exp\;\left(0.95\sqrt{Va}\right)$, where VA is the area-level variance.

Deviance Information Criterion (DIC), Akaike's information criterion (AIC), Bayesian's information criterion (BIC), and likelihood ratio were used to select the fitted model, and the model with a low information criterion value was an appropriate model. Based on these values, the final (model with individual and community-related variables) model has the smallest information criterion value among the model considered; therefore, the full model was best fitted. AOR with a 95% confidence interval in the multivariable model was used to select variables that have a statistically significant association with improved water sources and sanitation. All the data analyses were done using STATA Version 16.0 software.

### Ethical considerations

The data were accessed and utilized with the Central Statistical Agency of Ethiopia's prior permission. The first authors registered for dataset access and wrote the research topic, as well as its significance on the website. Then, we accessed the datasets at the website https://dhsprogram.com/Data/terms-of-use.cfm. The downloaded data were used simply for this study. As with all EDHS data, EMDHS 2019 data also were preserved as trustworthy of the participants in the survey.

## Results

### Descriptive statistics on socio-demographic characteristics

A total of 8,663 participants were involved in this study. The majority (72.62%) of household heads were men. Approximately 6,018 (69.47%) of the participants lived in a rural area. The highest proportion (47.65%) of educational status was no education, while the lowest (9.89%) was higher education. Nearly, 30% (30.53%) of study participants lived in urban. More than three-quarters (77.10%) of the included study subjects had no television (Table 1).

**Table 1:** Socio-demographic characteristics of study participants.

**Variables**	**Categories**	**Frequency (%)**	**Drinking water sources**	
**Individual-level variables**			Unimproved	Improved
Age of household head	<30	2,520 (29.09)	685 (7.91)	1,835 (21.18)
	30–40	2,287 (26.40)	675 (7.79)	1,612 (18.61)
	41–51	1,717 (19.82)	462 (5.33)	1,255 (14.49)
	>51	2,139 (24.69)	626 (7.23)	1,513 (17.47)
Minimum = 15, Maximum = 98, Std. Dev.= 16.49423				
Sex of household	Male	6,291 (72.62)	1,862 (21.49)	4,429 (51.13)
	Female	2,372 (27.38)	586 (6.76)	1,786 (20.62)
Educational status	No education	4,128 (47.65)	1,542 (17.80)	2,586 (29.85)
	Primary	2,715 (31.34)	693 (8.00)	2,022 (23.34)
	Second	963 (11.12)	136 (1.57)	827 (9.55)
	Higher	857 (9.89)	77 (0.89)	780 (9.00)
Wealth index	Poor	3,498 (40.38)	1,729 (19.96)	1,769 (20.42)
	Middle	1,285 (14.83)	379 (4.37)	906 (10.46)
	Rich	3,880 (44.79)	340 (3.92)	3,540 (40.86)
Having radio	No	6,170 (71.22)	2,012 (23.23)	4,158 (48.00)
	Yes	2,493 (28.78)	436 (5.03)	2,057 (23.74)
Having Television	No	6,679 (77.10)	2,380 (27.47)	4,299 (49.62)
	Yes	1,984 (22.90)	68 (0.78)	1,916 (22.12)
**Community-level variables**				
Media exposure	Unexposed	5,195 (59.97)	1,969 (22.73)	3,226 (37.24)
	Exposed	3,468 (40.03)	479 (5.53)	2,989 (34.50)
Level of education	Lower	4,308 (49.73)	1,808 (20.87)	2,500 (28.86)
	Higher	4,355 (50.27)	2,448 (28.26)	6,215 (71.74)
Poverty	Higher	4,276 (49.36)	1,902 (21.96)	2,374 (27.40)
	Lower	4,387 (50.64)	546 (6.30)	3,841 (44.34)
Residence	Urban	2,645 (30.53)	149 (1.72)	2,496 (28.81)
	Rural	6,018 (69.47)	2,299 (26.54)	3,719 (42.93)
Region	Tigray	714 (8.24)	189 (2.18)	525 (6.06)
	Afar	664 (7.66)	344 (3.97)	320 (3.69)
	Amhara	1,007 (11.62)	369 (4.26)	638 (7.36)
	Oromia	1,018 (11.75)	360 (4.16)	658 (7.60)
	Somali	657 (7.58)	355 (4.10)	302 (3.49)
	Benishangul-Gumuz	734 (8.47)	110 (1.27)	624 (7.20)
	SNNPR	1,017 (11.74)	336 (3.88)	681 (7.86)
	Gambela	693 (8.00)	182 (2.10)	511 (5.90)
	Harari	719 (8.30)	81 (0.94)	638 (7.36)
	Addis Ababa	702 (8.10)	10 (0.12)	692 (7.99)
	Dire Dawa	738 (8.52)	112 (1.29)	626 (7.23)

### Drinking water sources and sanitation

**Figure 1:**
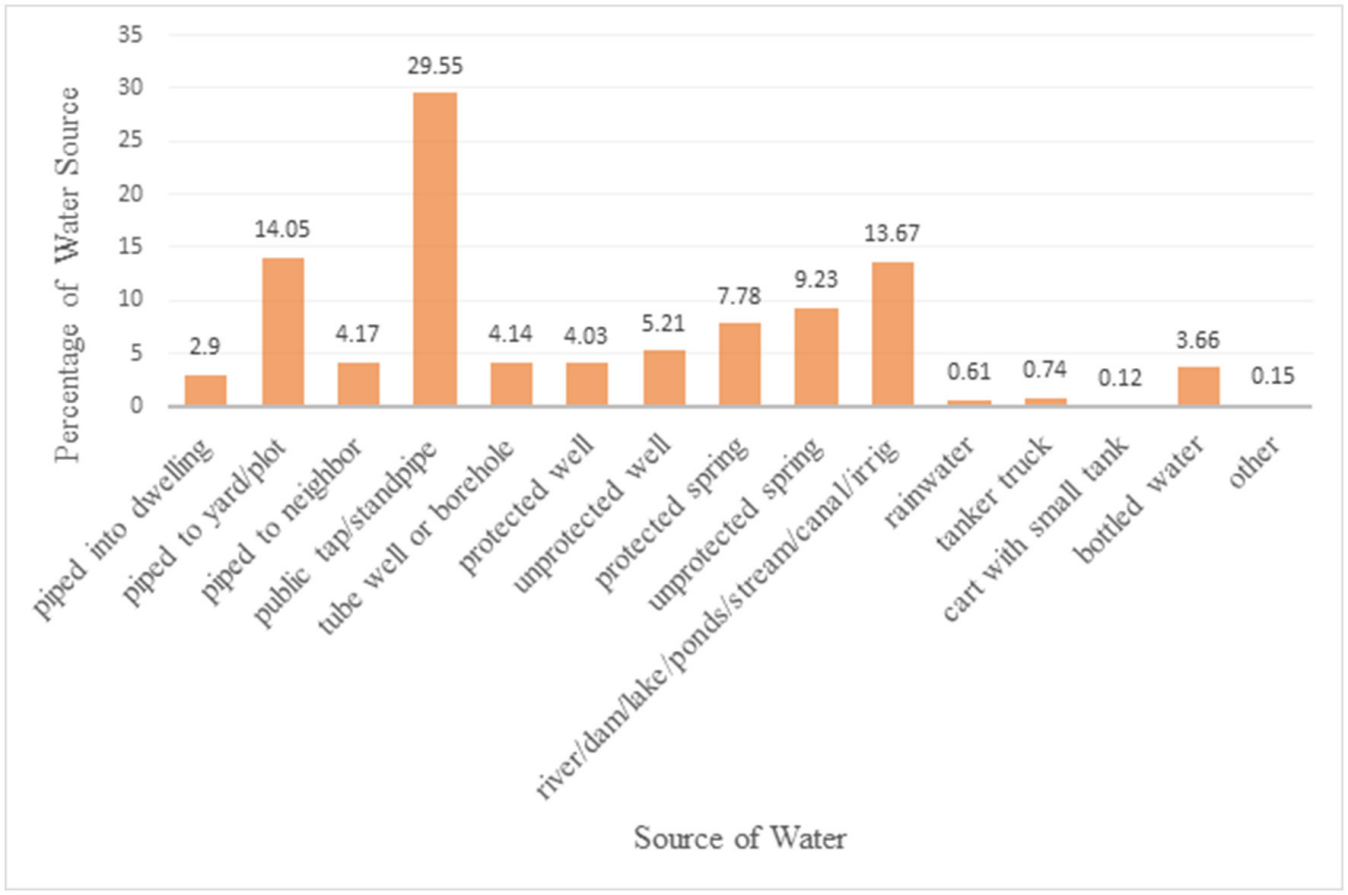
Proportion of different water sources based on the EMDHS 2019 datasets.

Figure 1 shows different types of water reported from EMDHS-2019 in the most recent survey in the country. Among the water source categories, public tap/stand pipe was the largest proportion (29.55%) followed by piped to yard/plot (14.05%) (Figure 1).

**Figure 2:**
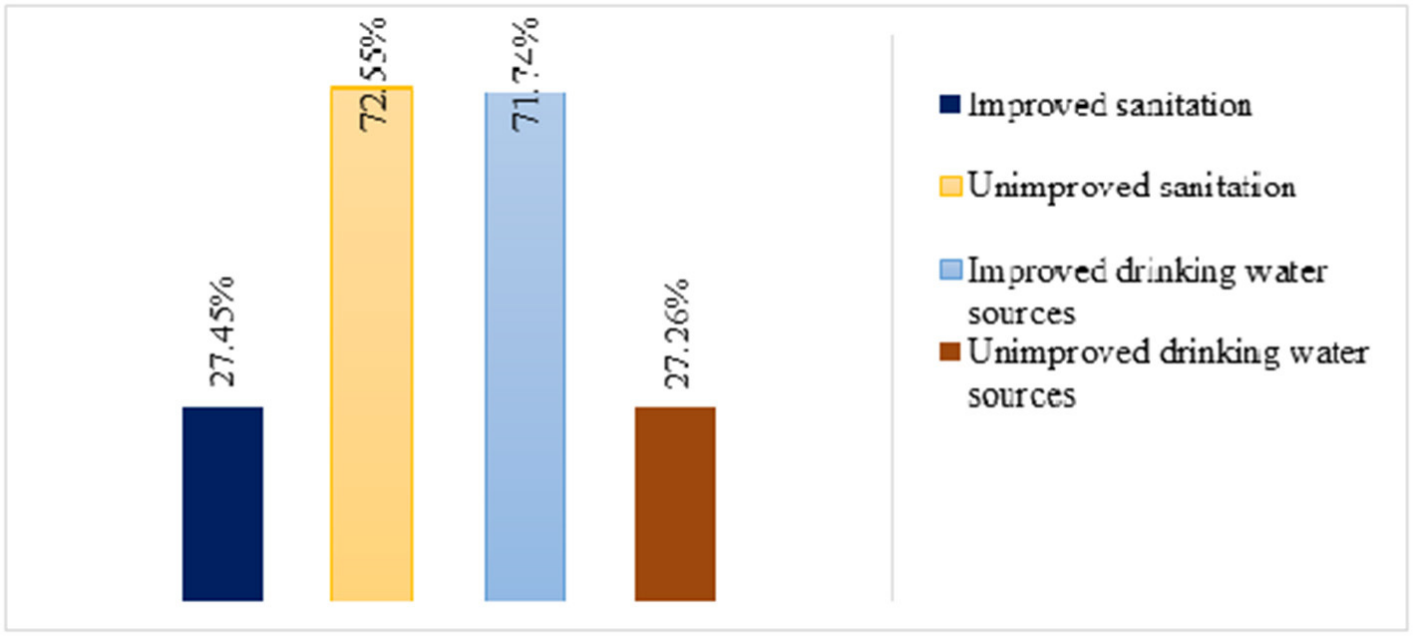
Level of improved water source and sanitation in Ethiopia from EMDHS 2019 Datasets.

The aforementioned water sources in Figure 1 are categorized as improved and unimproved water sources. Then, the overall national level of improved drinking water sources was 71.74% [95% CI = (70.78%−72.68%)]. While improved sanitation was 27.45% [95% CI = (26.52%−28.40%)], and unimproved sanitation was 72.55% [95% CI = (71.60%−73.48%)] (Figure 2).

**Figure 3:**
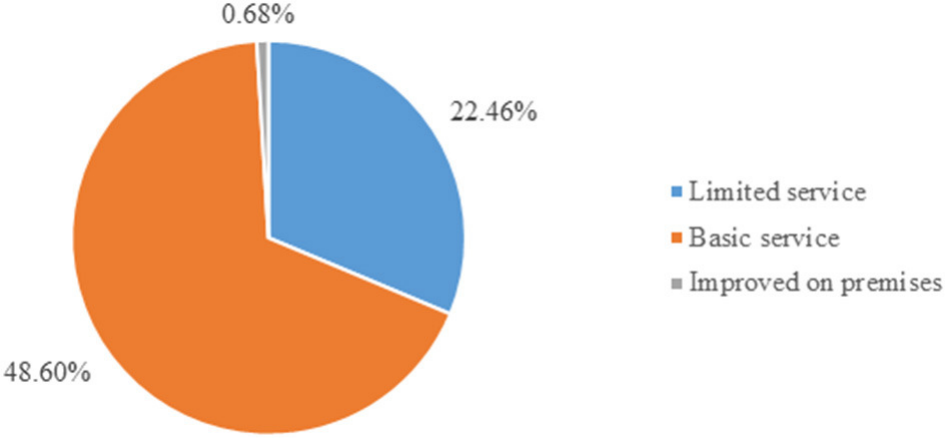
Improved water source classification based on time and improved source type in Ethiopia from EMDHS 2019 Datasets.

According to the WHO, there are three categories of improved water service: enhanced on premises, basic water service, and limited water service. In this study, only few (0.68%) of the participants accessed improved on premises services (Figure 3).

### Subgroup analysis of improved water and sanitation

**Figure 4:**
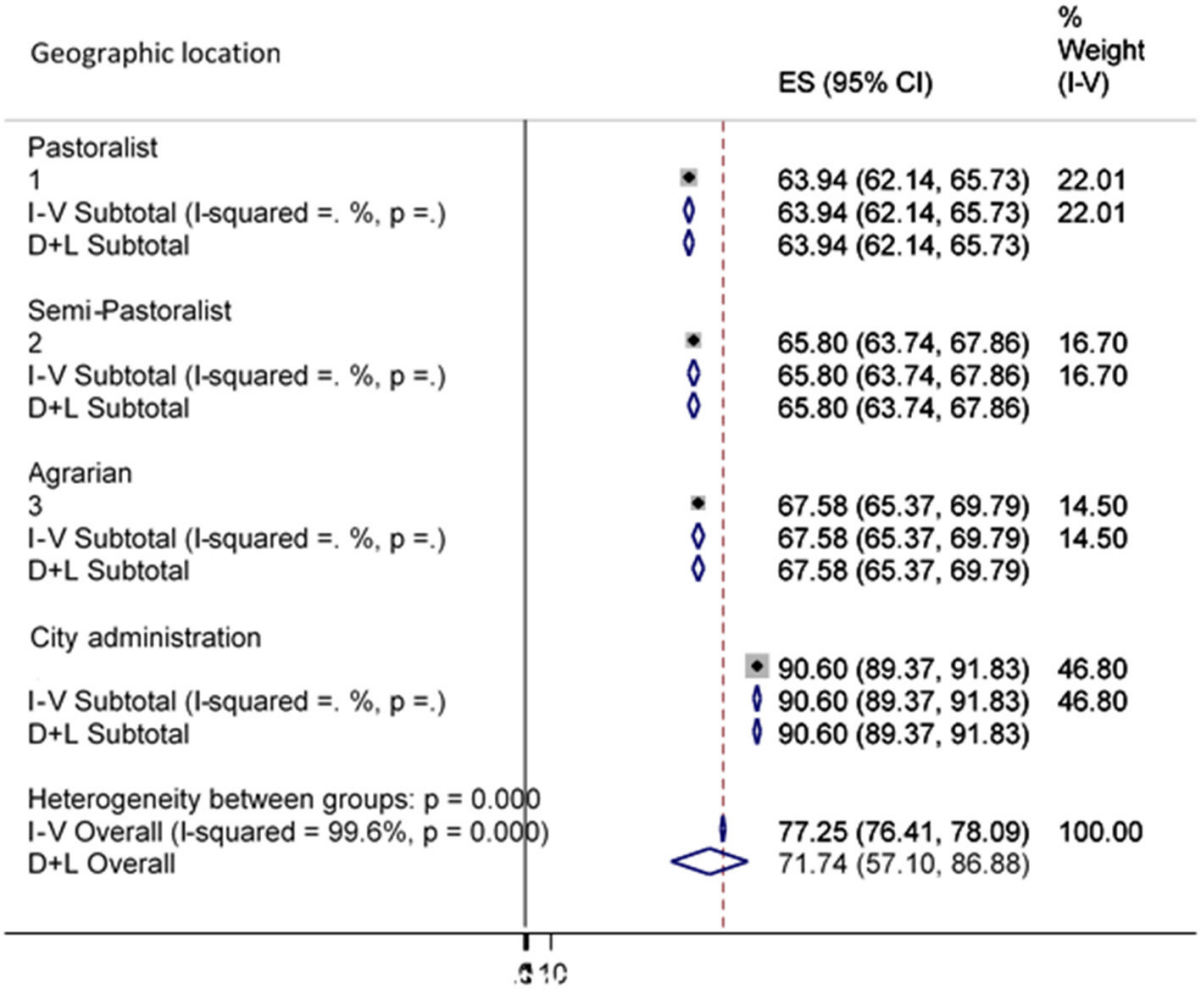
Sub-group analysis of improved water source by wealth status.

Figure 4 indicated that the subgroup analysis of the pattern for subgroup differences showed that there was a statistically substantial subgroup consequence based on wealth status (*p* < 0.001). The range of variation was from 42.52 to 96.89% between the poorest and the richest participants in accessing water from improved water sources (Figure 4).

**Figure 5:**
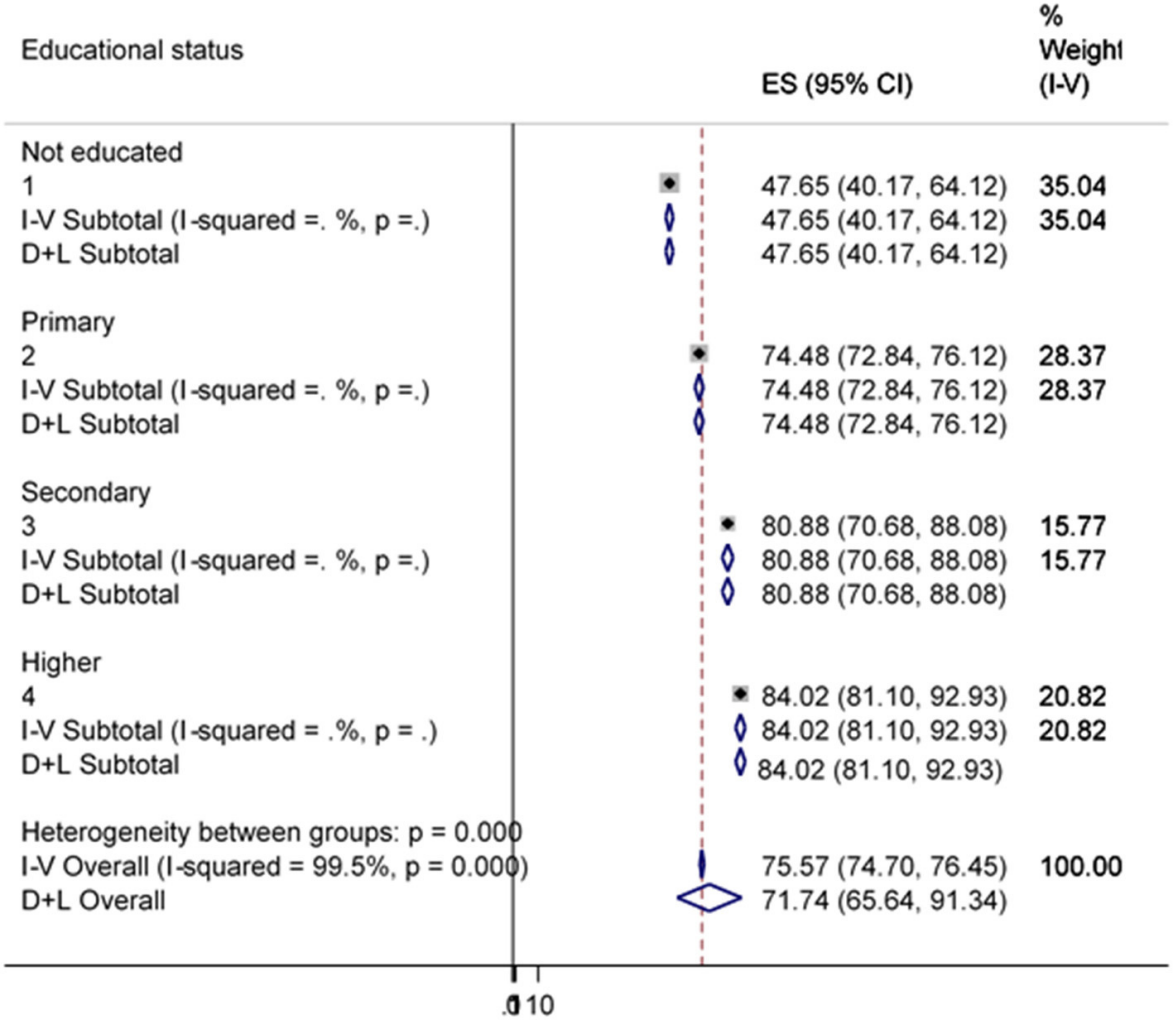
Sub-group analysis of improved water source by Geographic location.

Geographical location was another factor considered in the subgroup analysis. The outcomes of this subgroup analysis revealed a statistically important subgroup consequence (*p* < 0.001), which implies that the country's topography significantly changes the country's access to water from improved sources (Figure 5).

**Figure 6:**
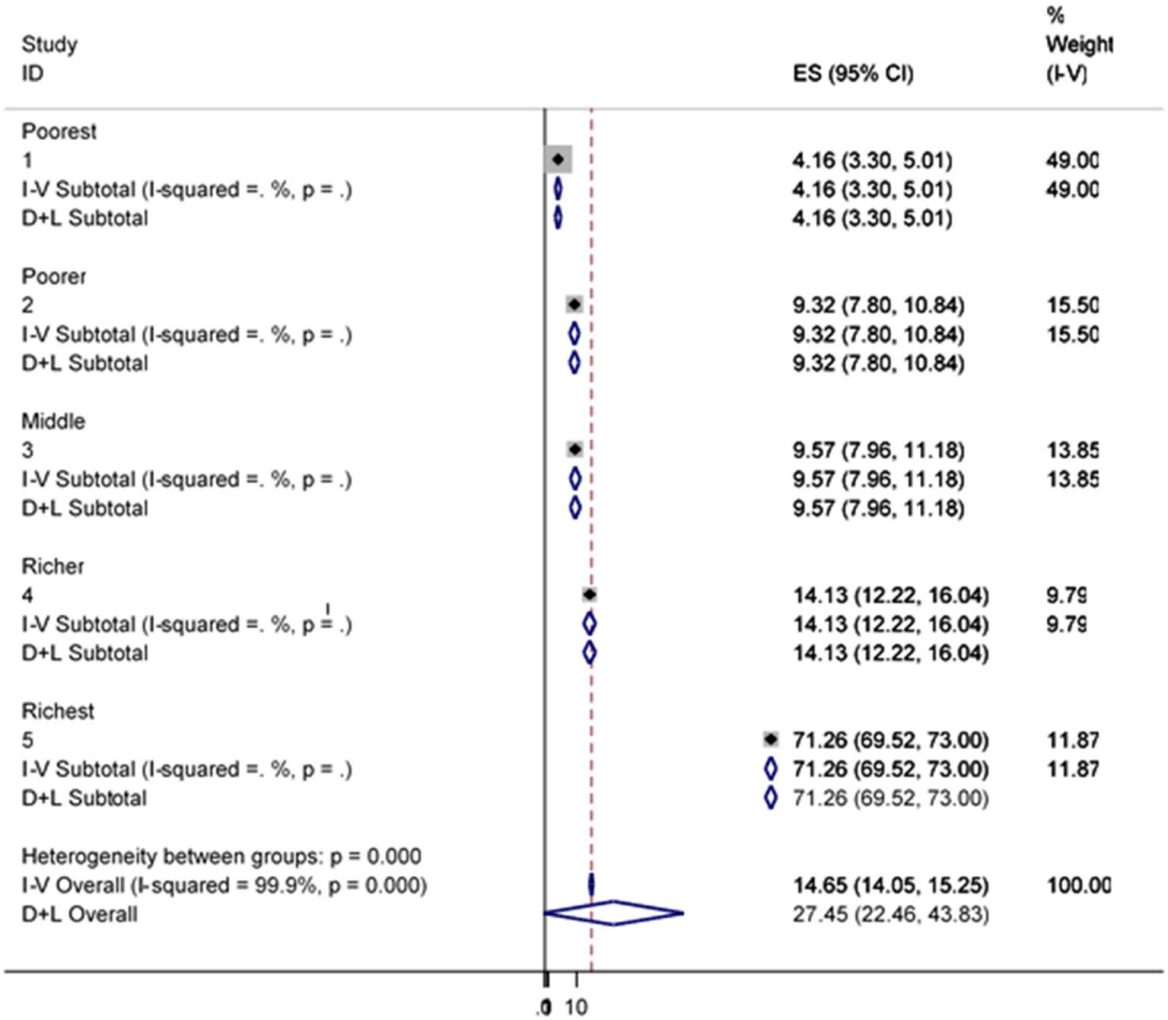
Sub-group analysis of improved water source by educational status.

Figure 6 indicates that there was a great heterogeneity among households in using improved water sources due to their variation in educational status (*p* = 0.001). This indicated that households owned by more educated people are more likely to have a variety of inputs and are more likely to use public assets such as piped water sources.

**Figure 7:**
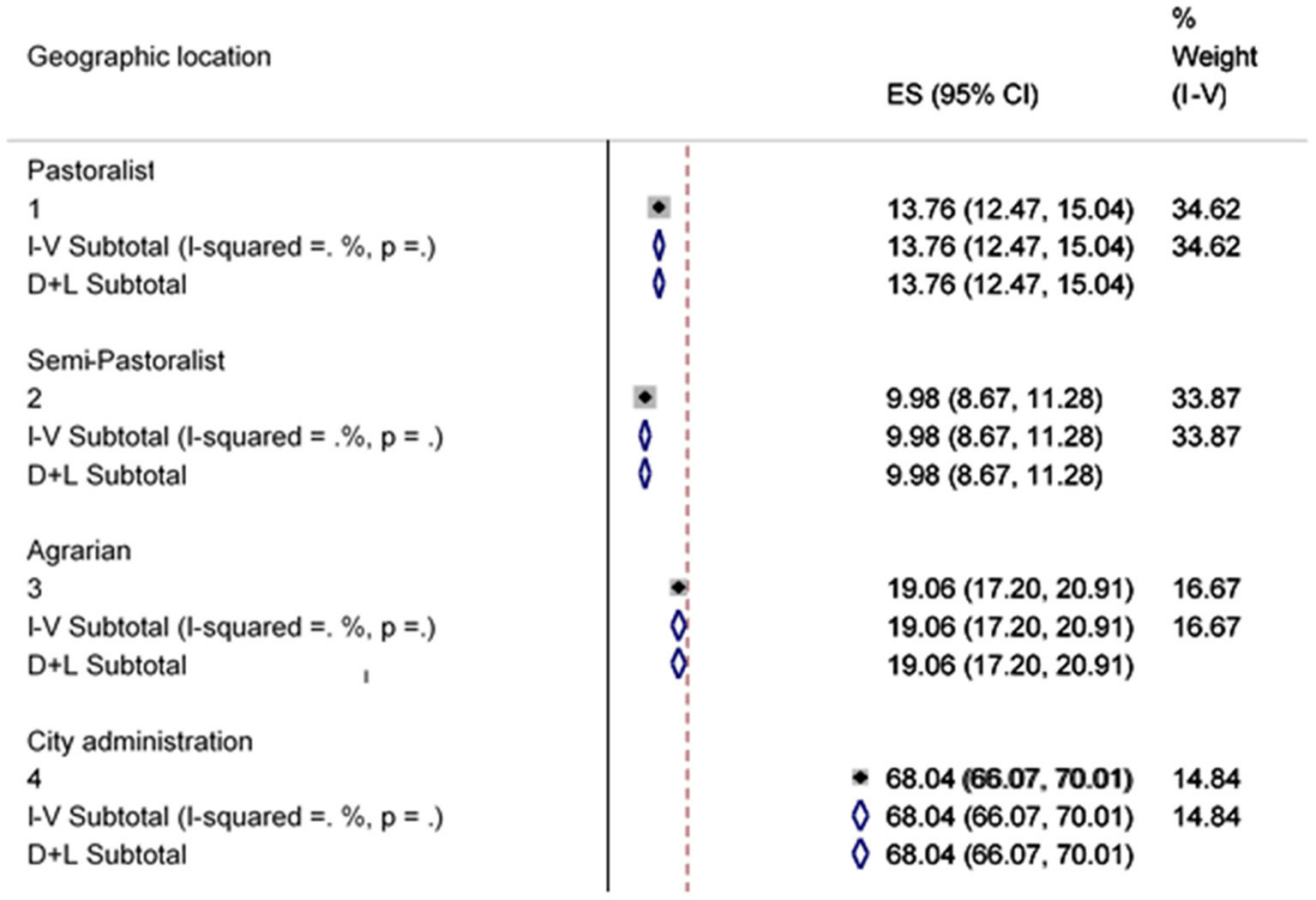
Sub-group analysis of improved sanitation facility by Wealth status.

The poorest lacks the monetary income to access improved sanitation facilities that support healthy lives. In this finding, there was great variation in having improved sanitation facilities among households due to their difference in wealth status (*p* = 0.001) (Figure 7).

**Figure 8:**
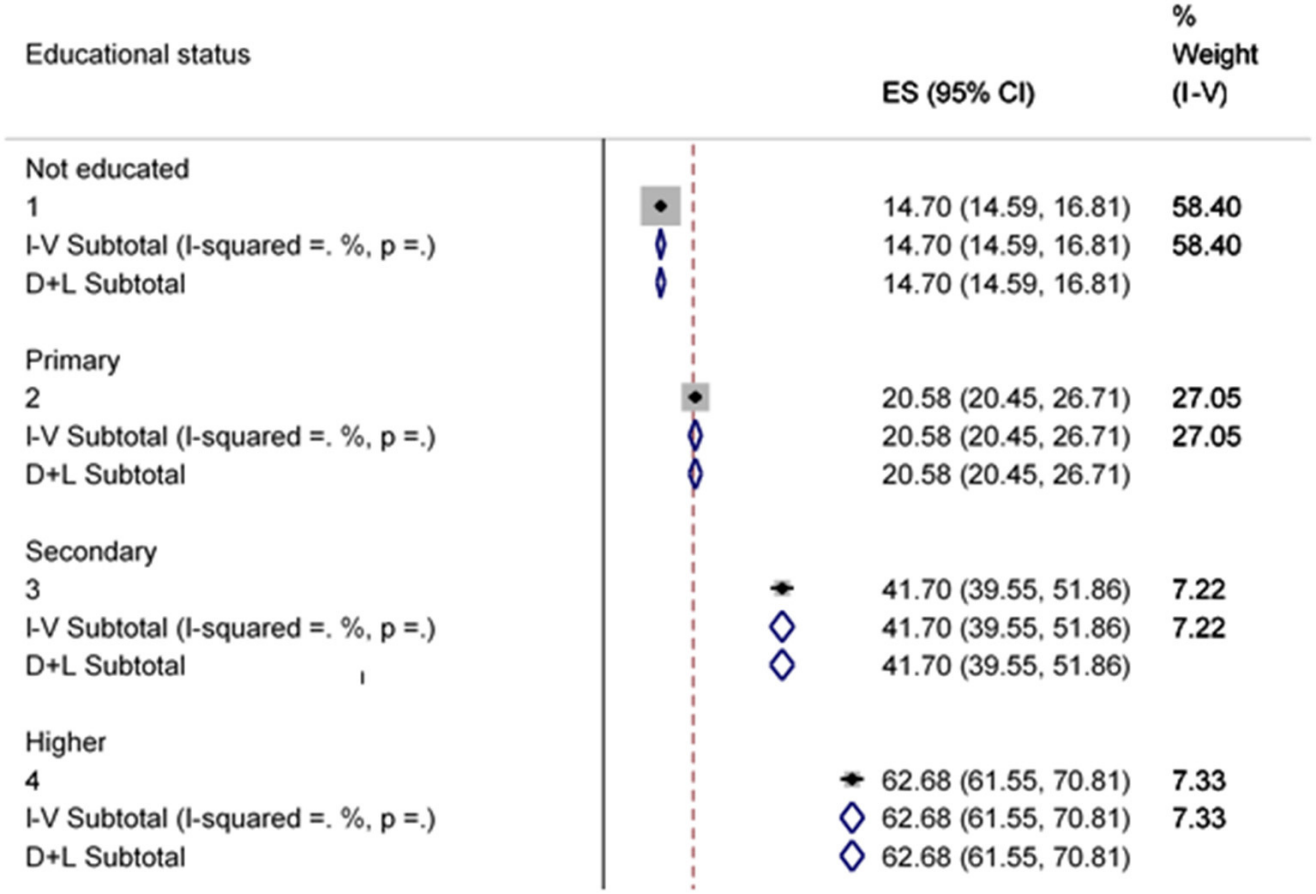
Sub-group analysis of improved sanitation facility by geographic location.

Figure 8 indicates that there was a great heterogeneity among households in using improved sanitation facilities due to their variation in areas where they live (*p* = 0.001). In more civilized areas, households are more likely to own various contributions and are more likely to benefit from them in public as long as they possess things like health education.

**Figure 9:**
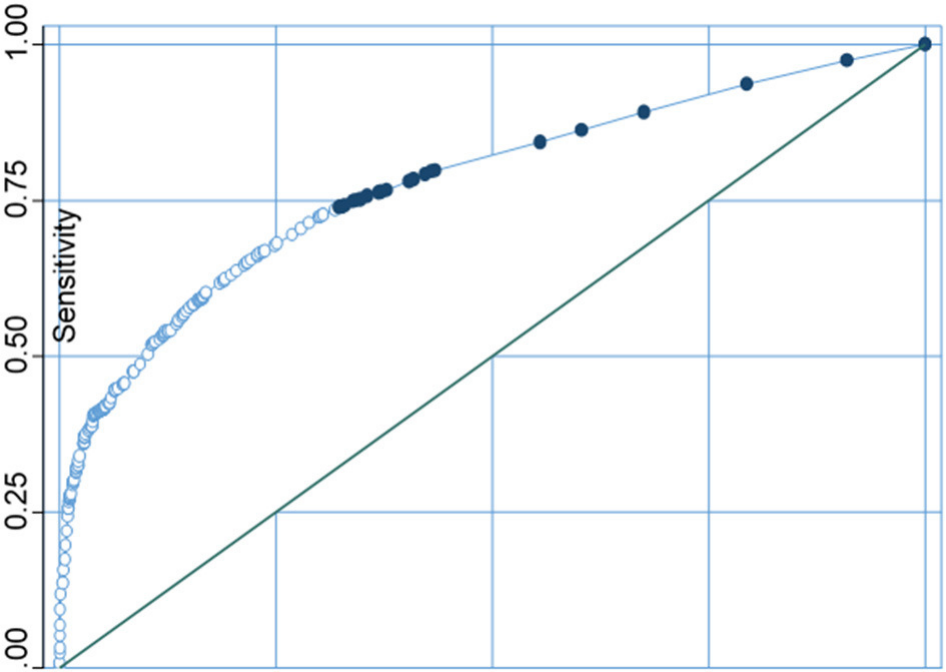
Sub-group analysis of improved sanitation facility by educational status.

There was a significant degree of heterogeneity among households in having improved sanitation facilities due to educational status differences (*p* = 0.001) (Figure 9).

### Imputation and specificity

Water source and available sanitation contain no soft missing (.) values, and the imputation variable is complete, finally, imputing nothing. Therefore, there was no influence of missing data on our conclusion of the results.

On the one hand, sensitivity, specificity, positive predictive value, and negative predictive value of the model were 84.41, 44.49, 79.42, and 52.92%, respectively. On the other hand, the sensitivity, specificity, positive predictive value, and negative predictive value of the model in sanitation were 65.01, 93.03, 77.92, and 87.54%, respectively (Table 2).

**Table 2:** Specificity analysis of unimproved water source and sanitation facilities.

**Classified**	**Unimproved water source**		
	**True**	**False**	**Total**
Correct	5,246	1,359	6,605
Incorrect	969	1,089	2,058
Total	6,215	2,448	8,663
	**Unimproved sanitation**		
	**True**	**False**	**Total**
Correct	1,546	438	1,984
Incorrect	832	5,847	6,679
Total	2,378	6,285	8,663

**Figure 10:**
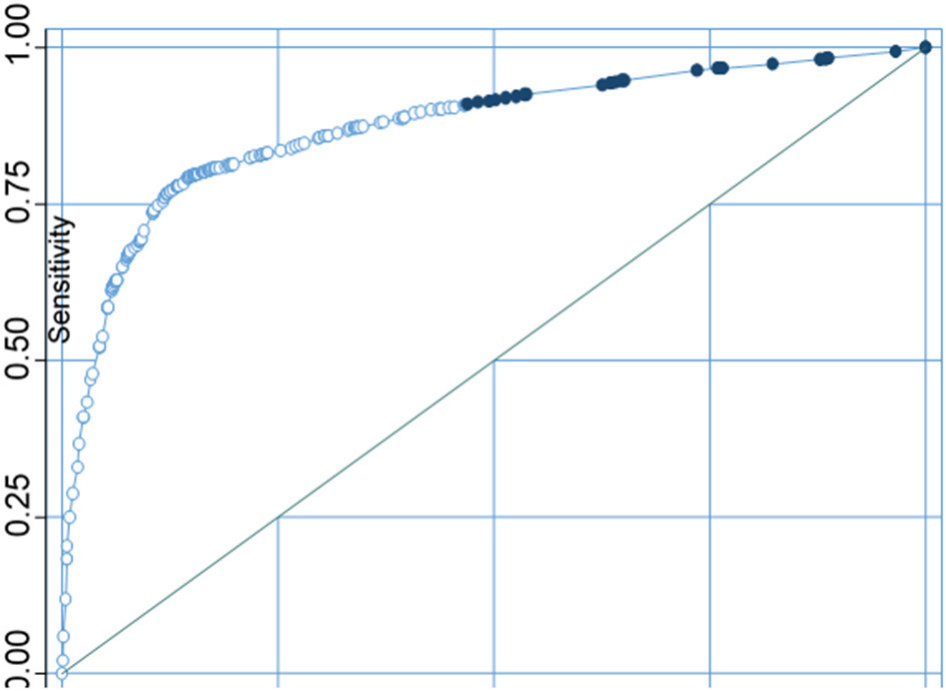
Sensitivity analysis curve of water sources.

The receiver operating characteristic (ROC) curve is a diagnostic method, shown as a graph that is used to assess the presentation of a binary logistic regression classification method. In this study, correctly classifying the water sources as improved and unimproved was 73.13%, and the area under the ROC curve was 0.7783 (Figure 10).

**Figure 11:**
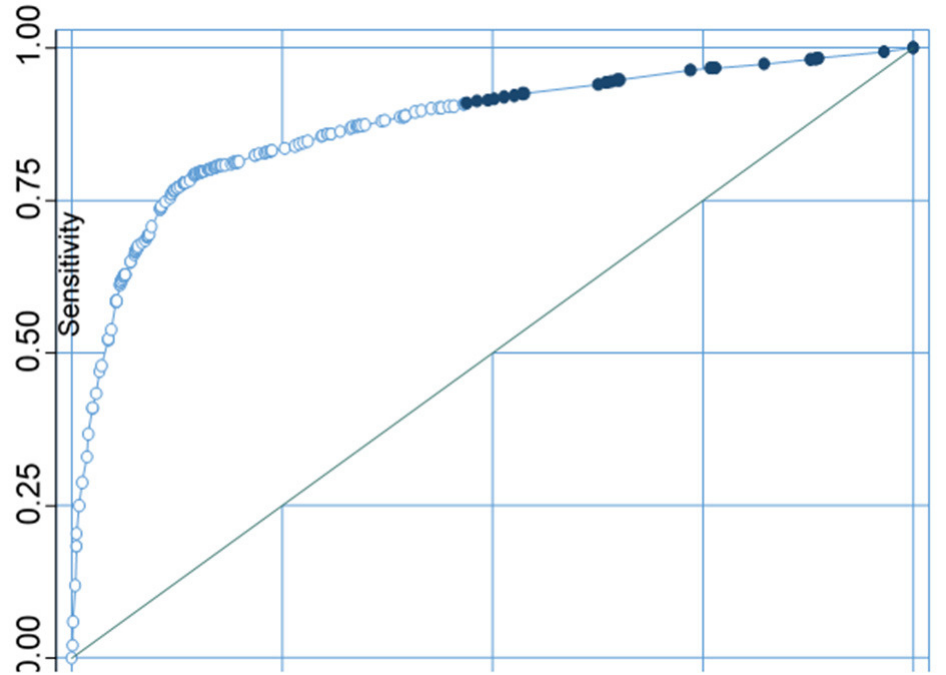
Sensitivity analysis curve of sanitation facilities.

Whereas, correctly classifying sanitation as improved and unimproved was 85.34%%, and the area under the ROC curve was 0.8726 (Figure 11).

### Determinants of household drinking water sources in Ethiopia using multilevel logistic regression analysis

Based on the final model results, wealth index, educational status, and having a television in individual-level variables, while community-level poverty, community-level education, community-level media exposure, and place of residence were statistically significant predictors of getting improved water sources.

The odds of getting water from an improved water source among the households with a middle level of wealth are 2.20 [AOR = 2.20; 95% CI (1.76–2.76)] times higher as compared with poor households. Whereas, the rich had a chance of 3.78 [AOR = 3.78; 95% CI (2.90–4.93)] times more likely to get water from an improved water source as compared with poor households.

The probability of getting water from an improved source among the households that had television was 1.85 [AOR = 1.85; 95% CI (1.49–2.95)] times more likely as compared with households that had no television.

Households in urban areas were 9.40 [AOR = 9.40; 95% CI (3.19–27.73)] times more likely to get water from improved sources as compared with rural area households.

The odds of getting water from an improved water source were 4.54 [OR = 4.54: 95% CI (1.95–10.53)] times more likely in households with higher education compared to households with a lower level of education.

The other predictor variable was community-level poverty. Households at lower-level poverty were 2.58 [OR = 2.58: 95% CI (1.10–6.03)] times more chance of getting water from improved sources compared to households with a higher level of poverty (Table 3).

**Table 3:** Multilevel regression analysis improved water source predictors variables in Ethiopia, EMDHS 2019.

**Variables**	**Model 0**	**Model I**	**Model II**	**Model III**
**Household head sex**				
Female		1.183 (0.98, 1.43)		1.15 (0.95, 1.39)
Male		1		1
**Wealth index**				
Poor		1		1
Middle		2.34 (1.86, 2.92)^*^		2.20 (1.76, 2.76)^*^
Rich		4.52 (3.48, 5.88)^*^		3.78 (2.90, 4.93)^*^
**Educational status**				
No education		1		1
Primary		1.74 (1.56, 1.94)^*^		1.52 (1.14, 1.834)^*^
Secondary		3.63 (2.99, 4.39)^*^		3.05 (2.32, 3.65)^*^
Higher		6.04 (4.74, 7.70)^*^		5.25 (3.87, 6.82)^*^
**Having television**				
No		1		1
Yes		2.07 (1.40, 3.08)^*^		1.85 (1.49, 2.95)^*^
**Having radio**				
No		1		1
Yes		0.95 (0.78, 1.16)		1.17 (0.59, 2.31)
**Residence**				
Rural			1	1
Urban			12.85 (4.41, 37.42)^*^	9.40 (3.19, 27.73)^*^
**Media exposure**				
Unexposed			1	1
Exposed			1.40 (1.17, 1.69)^*^	1.20 (1.25, 1.63)^*^
**Community-level education**				
Lower education			1	1
Higher education			5.14 (2.22, 11.94)^*^	4.54 (1.95, 10.53)^*^
**Community-level poverty**				
Higher			1	1
Lower			4.77 (2.06, 11.09)^*^	2.58 (1.10, 6.03)^*^
**Region**				
Pastoralist			1	1
Semi Pastoralist			0.35 (0.14, 1.88)	0.28 (0.13, 1.62)
Agrarian			1.07 (0.41, 2.80)	0.95 (0.36, 2.47)
City administration			2.23 (0.69, 7.23)	1.89 (0.58, 6.21)
VIF		1.73	1.45	2.81

1, Reference; ^*^p < 0.001; VIF, variance inflation factor.

### Measures of variation and model fit statistics

As shown in Table 3, the Null model indicated that there was substantial variation in getting water from the improved source across households due to their differences in the cluster. More than 80% (85.53%) of variation in getting water from the improved source is endorsed due to cluster variation. The MOR value from the Null model indicated that the median increased odds ratio of getting an improved water source if an individual moves to another area with an improved water source is available. The second model (Model II) revealed that the maximum PCV (34.61%) variations in getting water from the improved sources were due to individual-level factors. Model III showed the lowest values of AIC, DIC, and the large value of LLR (Table 4). Therefore, the final model was the best-fitted model.

**Table 4:** Measures of variation and model fitness for both improved water source and sanitation in Ethiopia.

**Parameters**	**Empty Model 0**	**Model I**	**Model II**	**Model III**
**Measures of variations for water sources**				
MOR	3.83	3.24	2.92	2.89
PCV	Reference	34.61%	19.55%	2.54%
ICC	0.8455	0.7816	0.7422	0.7373
Variance				
**Model fitness test statistics for water sources**				
AIC	5,535.568	5,365.221	5,393.423	**5,287.067**
BIC	5,549.702	5,414.689	5,457.024	**5,386.002**
Log likelihood	−2,765.7842	−2,675.6105	−2,687.7113	**−2,629.5334**
**Measures of variations for sanitation**				
MOR	2.95	2.05	1.45	1.59
PCV	Reference	54.47%	50.19%	6.10%
ICC	0.7566	0.5860151	0.4135	0.7373
**Model fitness test statistics for sanitation**				
AIC	5,867.791	5,552.671	5,542.386	**5,381.411**
BIC	5,881.925	5,623.339	5,605.988	**5,501.547**
Log likelihood	−2,931.8957	−2,766.3355	−2,762.1931	**−2,673.7055**

AIC, Akaike's information criterion; BIC, Bayesian's information criterion; ICC, intra-class correlation coefficient; PCV, variance partition coefficients.

Bold values = model fit.

### Factors associated with household improved sanitation in Ethiopia using multilevel logistic regression analysis

The final model results indicated that wealth index, educational status, and having television were individual-level variables, while community-level poverty, community-level education, community-level media exposure, place of residence, and region were community-level variables significantly associated factors with improved sanitation.

The odds of accessing improved sanitation among the households with a middle level of wealth were 1.86 [AOR = 1.86; 95% CI (1.38–2.49)] more times higher as compared with poor households.

While the odds of accessing improved sanitation among the households rich were 3.53 [AOR = 3.53; 95% CI (2.62–4.76)] times higher as compared with poor households.

The likelihood of having improved sanitation among the households with having a television was 2.31 [AOR = 2.31; 95% CI (1.49–2.95)] times more likely as compared with counterparts households that had no television.

Households in urban areas had the chance of accessing improved sanitation at 6.63 [AOR = 6.63; 95% CI (3.76–11.70)] times more likely compared to households in rural areas.

The odds of accessing improved sanitation are 2.43 [OR = 2.43: 95% CI (1.85–3.18)] times more likely in households with higher education compared to households with a lower level of education.

The other predictor variable was community-level poverty. Households in lower-level poverty were 2.32 [OR = 2.32: 95% CI (2.15–4.00)] times more chance of accessing improved sanitation compared to households with a higher level of poverty (Table 5).

**Table 5:** Multilevel regression analysis improved sanitation predictors variables in Ethiopia, EMDHS 2019.

**Variables**	**Model 0**	**Model I**	**Model II**	**Model III**
**Household head sex**				
Female		0.99 (0.98, 1.19)		0.94 (0.79, 1.12)
Male		1		1
**Wealth index**				
Poor		1		1
Middle		2.5 (1.52, 2.77)^*^		1.86 (1.38, 2.49)^*^
Rich		5.12 (3.83, 6.85)^*^		3.53 (2.62, 4.76)^*^
**Educational status**				
No education		1		1
Primary		1.08 (0.90, 1.29)^*^		1.04 (0.87, 1.25)
Secondary		1.31 (1.03, 1.68)^*^		1.23 (0.96, 1.57)
Higher		2.52 (1.92, 3.31)^*^		2.43 (1.85, 3.18)^*^
**Having television**				
No		1		1
Yes		2.54 (2.05, 3.16)^*^		2.31 (1.72, 3.10)^*^
**Having radio**				
No		1		1
Yes		1.13 (0.96, 1.34)		1.30 (1.00, 1.70)
**Residence**				
Rural			1	1
Urban			9.78 (5.50, 17.37)^*^	6.63 (3.76, 11.70)^*^
**Media exposure**				
Unexposed			1	1
Exposed			1.86 (1.57, 2.21)^*^	0.81 (0.57, 1.13)^*^
**Community-level education**				
Lower education			1	1
Higher education			1.86 (1.07, 3.09)^*^	2.30 (1.31, 5.53)^*^
**Community-level poverty**				
Higher			1	1
Lower			2.03 (1.19, 3.45)^*^	2.32 (2.15, 4.00)^*^
**Region**				
Pastoralist			1	1
Semi Pastoralist			0.54 (0.30, 0.99)^*^	0.55 (0.30, 1.00)
Agrarian			1.91 (1.05, 3.46)^*^	1.74 (0.97, 3.12)
City administration			6.09 (3.31, 11.20)^*^	5.02 (2.76, 9.12)^*^
VIF		1.35	1.56	2.65

1, Reference; ^*^p < 0.001.

## Discussion

Accessing waters from an improved source was moderate while accessing improved sanitation facilities was low.

Nearly 70% (71.74 %) of households' accessed drinking water from improved sources. This value was lower than the finding of the study on the world's population, which showed 74% of the households had access to a safe water source (27). This finding was similar to the study done in Benin in which 71.75% of the households accessed improved drinking water facilities (22). While this finding is higher than the studies done in Ethiopia (69.94%) (19), Zambian (64.5%) (28), Nepal (46.0%) (17), and Vietnam (64.6%) (29). In contrast, this finding is lower than the studies done in Ghana (88%) (20) Chi Linh, Viet Nam (86.5%) (30), and Minority Ethnic People in Vietnam (98.0%) (29). This difference could be due to the variation of study participants in socio-cultural, number of study participants, study design, period, and study setting. The largest proportion (48.60%) of improved water sources was basic water service, while the lowest (0.68%) was improved water on the premises due to the time taken for round trip. This might be due to the low expense for the development of the water sources as a possible, which enable improved water source for the community at large (25, 31).

More than one-fourth (27.45%) of the households accessed improved sanitation in Ethiopia. This finding was lower than the findings from Zambian (74.6%) (28), Kandahar City, Afghanistan (85.7%) (32), and a Rural Area of Haryana, North India (84.8%) (33). However, this finding was higher than the study done in Ghana (14% 0.06) (34). This variation may be attributed due to the difference in awareness in the study population, the government focuses on health policy, availability and accessibility of the services, economical differences in the countries, study design, study period, and sample size.

This evidence showed that the magnitudes of the households with access to the improved water source (from 69.94 to 71.74%) and improved sanitation facilities (from 25.36 to 27.45%) are nearly the same when compared with the previous EDHS 2016. However, in order to achieve worldwide access to at least basic drinking water and sanitation, it is expected to increase by many times the existing rates of improvement (3). Accordingly, reaching the SDG's aim to achieve Goal 6 Ensure access to water and sanitation for all by 2030 (35) will be challenging.

The final model results showed that wealth index, educational status, and having a television are individual-level variables, while community-level poverty, community-level education, community-level media exposure, and place of residence were statistically significant predictors of getting improved water sources and sanitation.

Wealth index and community level of poverty were statistical predictor variables of the type of water sources used at households. The current study revealed that middle and rich in wealth participants were more likely to have improved sources of drinking water services. This might be due to the increase in household income, households tend to use built improved sources of drinking water, whereas poor households are likely to stick with unimproved water sources. The household wealth index played a vigorous role in the achievement and utilization of improved toilet facilities because of the association between household wealth and access to improved wellbeing (36, 37).

The sex of the household head was not associated to have access to improved drinking water sources and sanitation. This finding contradicted with other previous studies (38–41).

The odds of accessing improved water sources and sanitation among households found in urban areas were more likely than in rural areas. This finding was supported by other previous studies (13, 28, 42–44). On average, households in urban areas access a higher level of improved sanitation service provision (20.48%) than households in rural areas (6.97%). The possible explanation for this variation might be the community in urban areas has a greater chance of accessing infrastructures, skilled labor, and technological resources, which enable improved sources of water and sanitation services.

Another socio-demographic significant factor was the education status and community level of education of the participant. The odds of getting water from improved sources and sanitation were increased among participants as their education level increased. This finding was consistent with a previous similar study that indicated that individuals with higher education need further levels of human resources and have a better developed empathy for the importance of access to safe drinking water to their health and wellbeing (27). This finding was supported by other previous studies (12, 20, 21). This association might be due to educated families possibly becoming informed on the benefits of using improved sources of drinking water. Alternatively, access to education is an important medium for indorsing consciousness toward using an improved source of water for better health.

Households who had television and community exposure to the media were more likely to have access to improved water sources and improved sanitation facilities in comparison with the households of their counterparts none exposed to media. A possible explanation for this could be continuous and informative media exposure might create possible health problems that could occur due to improved sanitation.

There were differences in accessing improved sanitation among regions. Households from the city administration were more likely compared to those from the pastoralists regions to have access to improved and improved sanitation services. This finding was supported by other previous similar studies (45). This variation might be due to that city administrations are with governmental organizations, have enough infrastructure for sanitation, and are aware of the community in sanitation. All these could lead to the households having improved sanitation for safeguarding their health and to create aesthetically attractive cities compared to pastoralist regions.

For both water source and type of sanitation facilities, model evaluation results points were found at the upper left junction of the ROC. A point projected by a pinpointing test falling into the area above the sloping represents a good investigative grouping, else a bad calculation. Thus, the models used in this study were appropriate for the sensitivity test.

## Conclusion

Generally, the level of accessing improved water sources is moderate but it lacks progress. While access improved, sanitation was lower. Wealth index, educational status, having television, community-level poverty, community-level education, community-level media exposure, and place of residence were statistically significant predictors of getting improved water source and sanitation. Based on these findings, great improvements should be made in providing access to the improved water source and sanitation facilities in Ethiopia. This can be done by creating awareness in a community of improved water sources and sanitation, through poverty reduction, an endowment to the poorest households, and communicating educator messages to the community using communication mediums. The finding of this study recommends that further efforts had better be made to escalate to get improved water sources and sanitation services among households found in rural areas. Health-related policy makers, local health administrators, NGOs, health extension workers, and all other stakeholders should work together in order to reduce health problems with WASH and to reach SDG's 6 goals.

## Data availability statement

Publicly available datasets were analyzed in this study. This data can be found here: https://dhsprogram.com/Data/terms-of-use.cfm.

## Author contributions

Data curation and methodology: JA and ME. Formal analysis and software: JA. Investigation and review and editing: JA, EA, AM, and ME. Validation and visualization: JA, EA, and ME. Writing: JA and AM. All authors contributed to the article and approved the submitted version.
